# HPLC-MS profiling and protective potential of the defatted aqueous methanol extract of two *Syzygium* species against cadmium chloride-induced nephrotoxicity in rats

**DOI:** 10.1371/journal.pone.0329586

**Published:** 2025-08-14

**Authors:** Fatma A. Moharram, Sahar S. Salem, Samah Shabana, Elsayed K. El-Sayed, Shimaa K. Mohamed, Mohamed A. Khattab, Kuei-Hung Lai, Asmaa A. Ahmed, Heba E. Elsayed

**Affiliations:** 1 Department of Pharmacognosy, Faculty of Pharmacy, Helwan University, Cairo, Egypt; 2 Department of Pharmacognosy, Faculty of Pharmaceutical Science and Drug Manufacturing, Misr University for Science and Technology, Giza, Egypt; 3 Department of Pharmacology and Toxicology, Faculty of Pharmacy, Helwan University, Cairo, Egypt; 4 Department of Cytology and Histology, Faculty of Veterinary Medicine, Cairo University, Giza, Egypt; 5 Graduate Institute of Pharmacognosy, College of Pharmacy, Taipei Medical University, Taipei, Taiwan; 6 PhD Program in Clinical Drug Development of Herbal Medicine, College of Pharmacy, Taipei Medical University, Taipei, Taiwan; 7 Traditional Herbal Medicine Research Center, Taipei Medical University Hospital, Taipei, Taiwan; Jadavpur University, INDIA

## Abstract

Cadmium (Cd), a highly toxic heavy metal, is used in food and agricultural products while displaying nephrotoxicity to animals and humans. The genus *Syzygium* (Myrtle family) is rich in precious phenolic metabolites with various therapeutic values. This study investigated the phenolic content and the therapeutic potential of the defatted 80% aqueous methanol extract (DE) of *S. malaccense* and *S. samarangense* leaves against Cd-induced kidney injury in rats for the first time. High-performance liquid chromatography-Mass spectrometry (HPLC-MS), in addition to Folin-Ciocalteu and aluminium chloride colourimetric methods, depicted the phenolic metabolites, total phenolic content, and total flavonoid content, respectively. The nephroprotective effect was investigated using fifty-six female *Sprague Dawley* rats divided into eight groups: control group, CdCl_2_-treated group (3 mg/kg/i.p/7 days), and three groups of each species treated with the DE (250, 500, and 1000 mg/kg/o.p., respectively). The phytochemical analysis revealed the richness of *S. samarangense* DE by phenolic and flavonoid content over *S. malaccense*. The HPLC-MS showed the tentative identification of sixty-two compounds, in positive and negative ionization modes, belonging to phenolic acids **(1−6**), flavonoids **(7−52),** and miscellaneous compounds **(53−62)**. Both extracts were considered safe up to 5 g/kg. At the maximum tested dose (100 mg/Kg), the DEs significantly (*p* < 0.001) boosted the levels of antioxidant markers by 3.3–6 fold, lessened the inflammatory indicators by 66.8%−75.1%, and reduced the apoptotic parameters by 45.4–73.3%, compared to the CdCl_2_-treated group. Additionally, the DEs maintained the mitochondrial function and inhibited autophagy *via* decreasing adenosine monophosphate-activated protein kinase by 49.2%−50.6%, and baclin-1 by 49.5%−56.1%. Additionally, the DEs increased the mammalian target of rapamycin (mTOR) by 4.7–4.9 fold. Additionally, the DE ameliorated CdCl2-induced elevations in serum ALT and AST, indicating a protective effect against systemic toxicity. Ultimately, the DE of *S. malaccense* and *S. samarangense* protect against Cd-induced nephrotoxicity that may be correlated to their abundant phenolic content. However, selecting suitable formulations and implementing clinical studies are among the future directions.

## Introduction

Cadmium (Cd) is a hazardous heavy metal renowned for its toxic potential to the kidneys [1]. Humans may be at risk from two types of Cd exposure. On top is the occupational Cd exposure through engaging with industrial activities [2] in addition to non-occupational exposure *via* contaminated food and water [3]. After Cd-exposure, multifactorial mechanisms are implicated in the pathogenesis of Cd-toxicity, such as oxidative stress, mitochondrial dysfunction, apoptosis, and inflammation [4–6]. Moreover, autophagy pathways are considered sensitive biomarkers for kidney damage upon Cd intoxication [7,8]. Currently, there is no standard therapy for Cd-induced nephrotoxicity, which underscores the urgent demand for the scientific community to discover a promising treatment, particularly given the growing global interest in the therapeutic values of antioxidants [9].

Phenolic compounds are a miscellaneous group of plant-derived metabolites that include different subclasses. They possess a unique chemical scaffold with potent antioxidant and anti-inflammatory activities [10–12]. Since oxidative stress and inflammation play essential roles in the prognosis of kidney diseases, phenolic metabolites offer potential as natural mediators that can modify these pathological processes [13–19]. Several plants are abundant in phenolics, including but not limited to *Syzygium* species (Myrtle family) [20,21]. Among the limitley explored species are *Syzygium malaccense* (L.) Merr. & L. M. Perry and *Syzygium samarangense* (Blume) Merr. & L. M. Perry. They are distributed in tropical and subtropical regions of Africa and Asia, and are renowned for their edible fruits [21–24]. In traditional medicine, *S. malaccense* was used as an anti-inflammatory, while the leaves and fruits were pharmacologically proven to possess anti-microbial and antioxidant activities, at least in part, due to their phenolic composition [25–31]. Extracts of *S. samarangense* displayed anti-inflammatory, analgesic, hepatoprotective, and diuretic effects [21,32]. These pharmacological activities are attributed to different classes of phenolic metabolites [21,33–35]. Although some biological activities were reported for *S. malaccense* and *S. samarangense*, their potential protective effect against Cd-induced nephrotoxicity has not been reported. Hence, encouraging our research team in the current study to investigate their nephroprotective potential.

Plant extracts represent a complex mixture of hundreds of diverse metabolites [36], making their analysis a challenging task. Although different chromatographic and spectroscopic approaches are available, high-performance liquid chromatography-mass spectrometry (HPLC-MS) permits comprehensive extract analysis on its own [37]. As a state-of-the-art analytical coupling approach, it merges the efficient separation aptitudes of HPLC with the supreme sensitivity and specificity of MS [37]. Hence, it provides valuable and prompt structural information on the metabolites detected without the need for isolation. Additionally, it is noticeable by its applicability to varied analyte types (ranging from small molecules to large polymers), providing higher resolution, greater sensitivity, and precision [36,37].

In the current study, we aimed to profile the phenolic content and composition of the defatted aqueous methanol extract of *S. malaccense* and *S. samarangense* using HPLC-MS. In addition, we evaluated their nephroprotective activity in a Cd-induced nephrotoxicity *in vivo* model.

## Materials and methods

### Plant material

*Syzygium malaccense* (L.) Merr. & L. M. Perry and *Syzygium samarangense* (Blume) Merr. & L. M. Perry leaves were supplied (June to August 2021) from Mazhar Botanical Garden, Cairo, Egypt. The plants were collected after the garden authorities’ permission, following the local garden`s guidelines for collection under the supervision of Dr. Trease Labib, a senior botanist at Mazhar Botanical Garden, who confirmed the species’ identity and the species’ names following the International Plant Name Index (IPNI). Samples from each species were kept in the Pharmacognosy Department, Faculty of Pharmacy, Helwan University under the numbers 01Sma, 2021, and 01Ssa, 2021. The plant material under study is endotoxin-free, and the study protocol was approved by the Ethics Committee of the Faculty of Pharmacy, Helwan University (Approval No: 11A2023).

### Chemicals and reagents

Methanol, *n*-hexane, normal saline, buffered formalin, and phosphate buffer were supplied from El Nasr Pharmaceutical Chemicals Co. (Gesr El Suez, Cairo, Egypt). Folin-Ciocalteu reagent, gallic acid, catechin, AlCl_3_, anhydrous cadmium chloride (CdCl_2_), ethanol, sodium thiopental, and hematoxylin-eosin staining solution were obtained from Sigma-Aldrich Inc. (St. Louis, MO, United States).

### Preparation of the defatted extract (DE)

*S. malaccense* (275 g) and *S. samarangense* (230 g) leaves were individually extracted with 80% aqueous methanol under reflux (3 x 4 L). The filtered, pooled extracts were evaporated under reduced pressure (60°C) to yield 35.0 g and 22.0 g of *S. malaccense* and *S. samarangense* dry extracts, respectively*.* The extracts were defatted by *n*-hexane under reflux (3 x 1 L) followed by solvent evaporation under *vacuum* using a rotary evaporator, followed by lyophilization to yield 10.0 g and 7.0 g (*n*-hexane) and 20.0 g and 10.0 g (defatted extract, DE) for *S. malaccense* and *S. samarangense,* respectively*.* The dried DE from each species was subjected to quantitative and qualitative phenolic profiling, and we investigated its protective potential in the Cd-induced nephrotoxicity *in vivo* model. The remainder of the extract was kept in a well-sealed container at −80°C.

### Estimation of total phenolic content (TPC)

It was estimated following the Folin-Ciocalteu colorimetric method [38]. The absorbance was determined at 725 nm on a UV-visible spectrophotometer (Jasco V-730, Jasco Corporation, Japan). The results were calculated from the calibration curve of the standard (gallic acid 50–300 μg/mL) using the following equation:


$$y=0.0237x+0.0455\;(R^{2}=0.9976)$$

The results were stated as gallic acid (mg) equivalent (mg GAE/mg) of dry weight.

### Estimation of total flavonoid content (TFC)

It was measured colorimetrically using the AlCl_3_ [38]. The absorbance was measured at 510 nm on a UV-visible spectrophotometer (Jasco V-730, Jasco Corporation, Japan). The results were deduced from the equation of the calibration curve constructed from the standard (catechin, 50–300 μg/mL) as follows:


$$y=0.0088x+0.0430\;(R^{2}=0.9994)$$

Results were expressed as milligrams of catechin equivalent (mg CE/mg of dry weight).

### Determination of phenolic profile using HPLC-MS

The HPLC-MS analysis was performed on the XEVO® triple quadrupole (TQD) mass system (Waters™ Corporation, Milford, MA, United States) operated in negative and positive ionization modes. Chromatographic separation was carried out on the ACQUITY UPLCBEH C18 column (1.7 µm x 2.1 mm x 50 mm, 28.4 °C) using 0.1% formic acid in H_2_O as solvent A and MeOH as solvent B. Isocratic gradient elution was implemented using a flow rate of 0.2 mL/min for 32 min as follows: 10% B for 5 min, 30% B for 10 min, 70% B for 7 min, then 90% B for 10 min. The MS acquisition range was *m/z* 50–1000.

### *In vivo* nephrotoxic activity induced by cadmium chloride (CdCl_2_)

#### Experimental animals.

Adult female Sprague-Dawley rats (9–10 weeks old, 180–200 g) were purchased from 223 VACSERA (Helwan, Cairo, Egypt). Rats were housed (3–4 rats/cage) at a controlled 224 temperature of 22°C ± 3°C with a 12-hour light/dark cycle. Standard rodent chow and water were provided ad libitum. All procedures were conducted following the ethical standards approved by the Ethics Committee of the Faculty of Pharmacy, Helwan University (Approval No: 11A2023). The study was conducted in compliance with the ARRIVE guidelines 2.0, the EU Directive 2010/63/EU for the protection of animals used for scientific purposes, and the NIH Guide for the Care and Use of Laboratory Animals (8th edition). The experimental design included the possibility of mortality due to the nature of the pharmacological intervention. Therefore, animals were monitored at least twice daily for clinical signs of pain, distress, or illness, including reduced mobility, abnormal posture, respiratory distress, piloerection, and failure to groom. If any animal exhibited signs of severe distress or met predetermined humane endpoints (e.g., > 20% weight loss, loss of appetite > 48 hours, or unresponsiveness), it was euthanized immediately via an overdose of sodium thiopental administered intraperitoneally (≥150 mg/kg). No animals died unexpectedly, and euthanasia was performed according to ethical guidelines to minimize suffering. All efforts were made to refine procedures and reduce the number of animals used. The Humane endpoints checklist is supplied as supporting material S1.

#### Acute toxicity study.

We used adult female rats, strain *Sprague Dawley* (180–200 g, n = 6), to establish the acute study. In a dose-dependent approach, the rats were administered *S. malaccense* DE and *S. samarangense* DE at 50 mg/kg, 200 mg/kg, 500 mg/kg, 1g/kg, 2.5 g/kg, and 5 g/kg. The rats were well observed for 24 h for any symptoms of toxicity, side effects, behavioral changes, movement patterns, diarrhea, and death.

#### Experimental design.

Fifty-six rats were randomly divided into eight groups (n = 7). The control group received 0.9% saline solution as a vehicle for seven days. In the CdCl_2_ group (group 2), rats were injected with CdCl_2_ (3 mg/kg/ intraperitoneal) for 7 days [39]. Animal groups 3–5 were treated for seven days with the DE of *S. malaccense* at 250, 500, and 1000 mg/kg orally, about one hour before the administration of CdCl_2_. On the other hand, groups 6–8 were treated for seven days with the DE of *S. samarangense* at 250, 500, and 1000 mg/kg doses, respectively, about one hour before the administration of CdCl_2_. On the 8^th^ day, rats were intraperitoneally injected with sodium thiopental (40 mg/kg) [40] to induce anesthesia. Blood samples were collected from the retro-orbital plexus using non-heparinized tubes. Rats were euthanized by cervical dislocation, and the kidneys were isolated, washed with cold saline, and weighed. The right kidneys were homogenized in an ice-cold phosphate buffer (10 mM, pH 7.4) to prepare tissue homogenates of 10% w/v for biochemical parameters. The left kidneys were preserved in formal saline (10%) for histopathological analysis.

### Measurements of biochemical markers

#### Serum urea and creatinine.

Urea and creatinine levels were determined using colorimetric kits (Catalog #K375-100 and K625-100, respectively) purchased from BioVision (Milpitas, United States). The colour intensity was measured on a Jenway™ 7315 spectrophotometer (Fisher Scientific, Leicestershire, UK).

#### Oxidative stress parameters.

The levels of GSH and SOD in the kidney tissue homogenates were quantified using the colorimetric kit (Catalog #K464-100 and K335-100 respectively) obtained from BioVision (Milpitas, United States). The colour intensity was measured on a Jenway™ 7315 spectrophotometer (Fisher Scientific, Leicestershire, UK).

#### Inflammatory parameters.

Using specific ELISA kits, the levels of TNF-*α* (Catalog # 438204, BioLegend, San Diego, United States), IL-1*β* (Catalog # E0119Ra, Bioassay Technology Laboratory, Shanghai, China), and NF-κB p65 (Catalog # ABIN1380657, Antibodies-online, Canada) were quantified in kidney tissue homogenates using NS 100 microplate reader (Heruvan Lab Systems, Selangor, Malaysia).

#### Apoptotic parameter.

The level of the apoptotic marker, caspase-3, was determined in the kidney tissue homogenates using an ELISA kit (Catalog # E4592-100, BioVision, Milpitas, United States) and NS 100 microplate reader (Heruvan Lab Systems, Selangor, Malaysia).

#### Mitochondrial dysfunction parameter.

The ATP level in kidney tissue homogenates was estimated using a colorimetric Kit (Catalog # ab83355, Abcam, Cambridge, United Kingdom). The colour intensity was measured on a Jenway™ 7315 spectrophotometer (Fisher Scientific, Leicestershire, UK).

#### Autophagic parameters.

For the determination of autophagic parameters, ELISA Kits for the phosphorylated mammalian target of rapamycin (p-mTOR) (Catalog # ab279869, Abcam, Cambridge, United Kingdom), Beclin-1 (Catalog# ER0416, FineTest, Wuhan, China), and phosphorylated adenosine monophosphate-activated protein kinase (p-AMPK) (Catalog # ER0730, FineTest, Wuhan, China) were used according to the manufacturer’s procedures. In brief, samples were added to their corresponding microtiter plate wells coated with the targeted monoclonal antibodies. Accordingly, any rat p-mTOR, beclin-1, or p-AMPK in the samples would bind to their corresponding immobilized antibodies. The wells were washed, and biotin-conjugated anti-rat p-mTOR, beclin-1, or p-AMPK antibodies (1:100) were added to their corresponding plates. The plates were washed, and avidin-horseradish peroxidase (avidin-HRP) was added. Then, the wells were washed, and tetramethylbenzidine (TMB) substrate solution was added to produce a blue color directly proportional to the amount of the detected proteins in the sample. The reaction was terminated by adding the stop solution, which changed the color from blue to yellow, and the intensity of the color was measured at 450 nm using an NS 100 microplate reader (Heruvan Lab Systems, Selangor, Malaysia).

#### Assessment of liver enzymes.

To evaluate the systemic toxicity of cadmium, the serum levels of alanine aminotransferase (ALT) and aspartate aminotransferase (AST) were measured using Biomatik ELISA kits (Cat no: EKU02211, EKE62019, Kitchener, Ontario, N2C 1N6, Canada, respectively). The procedures were carried out according to the manufacturer’s instructions

#### Histopathological analyses.

Kidney tissue samples from the investigated groups were fixed in 10% neutral buffered formalin for three days. The samples underwent a series of ethanol dehydration steps, followed by xylene clearing and embedding in Paraplast embedding media. Kidney tissue sections (4 µm) were cut using a rotary microtome and then fixed on glass slides for further examination. Finally, using hematoxylin and eosin, tissue slices were stained and inspected under a light microscope [41].

### Statistical analysis

All experiments were implemented in triplicate, and values were represented as mean ± SEM. Results were analyzed using ANOVA followed by Tukey’s test, while the student’s t-test was implemented to analyze the TPC and TFC data. The Prism software, version 8, was used for the statistical analysis (GraphPad Software Inc., San Diego, United States). *P*-value, which is less than 0.001, is statistically significant and denoted with an asterisk or a letter, as indicated.

## Results

### Extraction, quantification, and identification of phenolic metabolites

#### Quantification of phenolic and flavonoid contents from two *Syzygium* species.

As stated earlier, the total phenolic and flavonoid contents in the DE of the two investigated species were measured using colourimetric assays. The results (Table 1) have shown that *S. samarangense* possesses a TPC that is 1.5-fold higher than *S. malaccense,* and the difference is statistically significant. Interestingly, this is the first study reporting the TPC for *S. samarangense.* However, it has been previously reported from *S. malaccense* by several researchers, notably ranging from 62.31 ± 8.32 to 88.73 ± 7.71 mg GAE/g [25,30]. On the other hand, *S. samarangense* showed significantly higher total flavonoid content than *S. malaccense* by 1.2-fold. This is the first report on the TFC of *S. samarangense,* although it has been quantitatively documented before (8.10 ± 46.75 CE/g) by Batista et al. [25]. The possible deviation in our data from those reported in the literature could be attributed to the geographical collection site’s impact and the variations in the extraction conditions (solvent, time, and temperature) [42].

**Table 1:** Estimated total phenolic content (TPC), and total flavonoid content (TFC) in the defatted extract (DE) of *S. malaccense* and *S. samarangense* leaves.

Species	TPC (mg GAE/g)	TFC (mg CE/g)
***S. malaccense***	84.24 ± 0.94	6.41 ± 0.36
***S. samarangense***	123.70 ± 1.41*	7.83 ± 0.25*

Data represented as mean ± SEM of n = 3. *: significant from *S. malaccense (p* < 0.001).

#### HPLC-MS analysis for defatted extract (DE) of *S. malaccense* and *S. samarangense* leaves.

**Fig 1:**
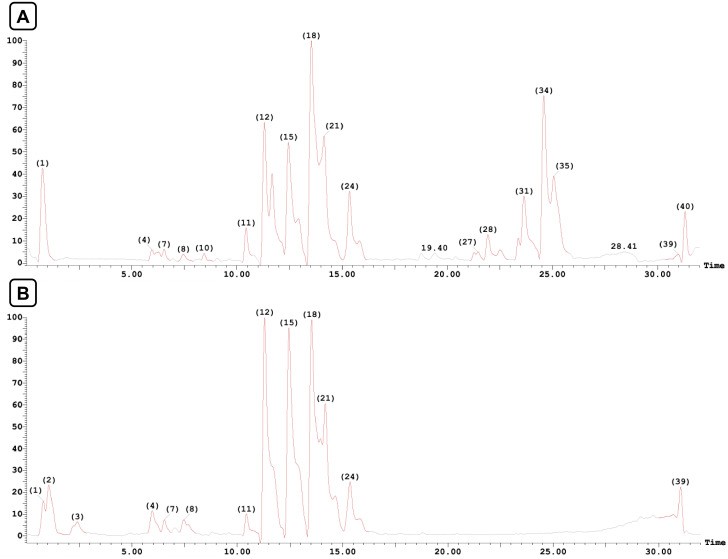
Total ion chromatogram (TIC) obtained from HPLC-MS of the defatted aqueous methanol extract of *S. malaccense* leaves in (A) positive and (B) negative ionization modes.

**Fig 2:**
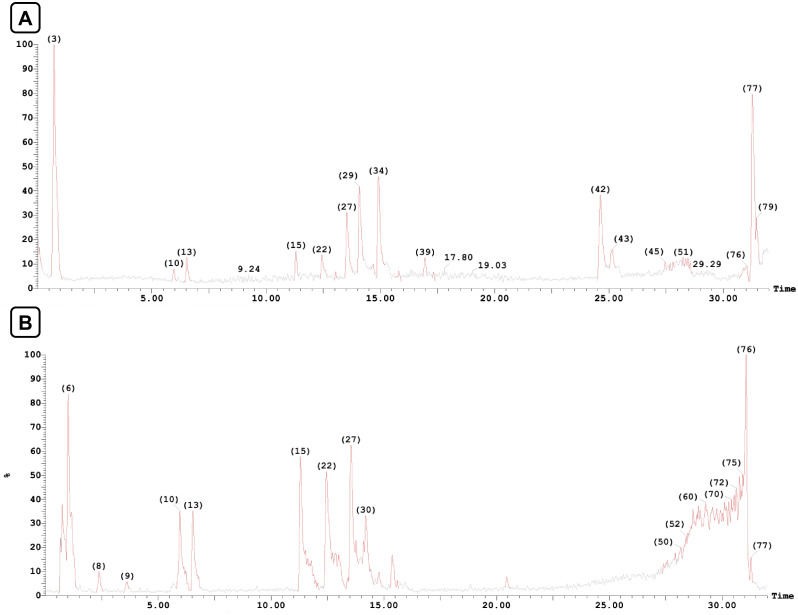
Total ion chromatogram (TIC) obtained from HPLC-MS of the defatted aqueous methanol extract of *S. samarangense* leaves in (A) positive and (B) negative ionization modes.

The HPLC-MS for the DE of the two *species* revealed the presence of sixty-two secondary metabolites belonging to various classes, which were tentatively identified in positive and negative modes (Table 2). Thirty-nine were detected in *S. malaccense*, while forty-six were in *S. samarangense*. The HPLC-MS chromatograms of the two species are displayed in Figs 1 and 2.

**Table 2:** HPLC-MS tentative identification of phenolic metabolites from the defatted extract (DE) of *S. malaccense* (SM) and *S. samarangense* (SS) leaves.

No.	Identified phenolics	Molecular formula	Retention time (min)	Mode of ionization	Molecular weight	Observed (*m*/*z*)	*SM*	*SS*	Reference
	**Phenolic acids**								
1	Gallic acid	C_7_H_6_O_5_	1.06	[M-H]^–^	170.12	169.0373	+	+	[32]
2	Methyl gallate	C_8_H_8_O_5_	2.41	[M-H]^–^	184.15	183.0698	+	+	[55]
3	Ellagic acid glucoside	C_20_H_16_O_13_	5.96	[M-H]^–^	464.333	463.1513	–	+	[49]
4	Hydroxycaffeic acid	C_9_H_8_O_5_	6.19	[M+H]^+^	169.157	197.1579	+	–	[56]
5	Ellagic acid	C_14_H_6_O_8_	6.21	[M-H]^–^	302.193	301.0638	–	+	[49]
6	Gallic acid glucoside	C_13_H_16_O_10_	6.54	[M+H]^+^	332.26	333.1248	+	+	[49]
	**Flavonoids Flavonol**								
7	Myricetin	C_15_H_10_O_8_	5.96	[M-H]^–^ [M+H]^+^	318.2370	317.1821 319.0877	+	+	[35]
8	Myricetin- *O*-rhamnoside	C_21_H_20_O_12_	5.97	[M-H]^–^ [M+H]^+^	464.3790	463.2279 465.1994	+	+	[46]
9	Isoquercetin	C_2_1H_20_O_12_	6.13	[M-H]^–^	464.376	463.1943	–	+	[25]
10	Quercetin	C_15_H_10_O_7_	6.13	[M-H]^–^	302.236	301.0468	+	+	[46]
11	Hyperin	C_21_H_20_O_12_	6.21	[M-H]^–^	464.376	463.1249	–	+	[52]
12	Isorhamnetin-*O*-rhamnoside	C_22_H_22_O_11_	6.29	[M+H]^+^	462.4	463.1954	+	–	[46]
13	Isorhamnetin-*O*-glucoside	C_22_H_22_O_12_	6.52	[M-H]^–^	478.403	477.1945	+	+	[25]
14	Mearnsetin	C_16_H_12_O_8_	6.52	[M+H]^+^	332.26	333.1245	+	+	[46]
15	Quercitrin	C_21_H_20_O_11_	6.54	[M-H]^–^	448.377	447.2192	+	–	[46]
16	Kaempferol –*O* glucoside	C_21_H_20_O_11_	6.54	[M-H]^–^	448.377	447.2192	+	–	[25]
17	Quercetin 4′-*O*-glucuronide	C_21_H_18_O_13_	6.54	[M-H]^–^	478.36	477.2255	+	+	[49]
18	Mearnsitrin	C_22_H_22_O_12_	6.79	[M-H]^–^	478.4	477.2544	+	+	[46]
19	Kaempferol	C_15_H_10_O_6_	14.56	[M+H]^+^	286.2390	287.1558	+	+	[35]
20	Quercetin 3-*O*-acetyl-galactoside)-*O*-rhamnoside	C_29_H_32_O_17_	24.21	[M+H]^+^	652.1622	653.4450	+	–	[57]
21	Quercetin 3’-sulfate	C_15_H_10_O_10_S	27.80	[M+H]^+^	382.3	383.4042	–	+	[58]
	**Flavone**								
22	3,4’,7-Tetrahydroxyflavone	C_15_H_10_O_6_	14.79	[M-H]^–^	285.0455	285.1601	–	+	[49]
23	Diosmin	C_28_H_32_O_15_	21.29	[M+H]^+^	608.549	609.4222	+	–	[49]
24	Apigenin-7-O-diglucuronide	C_27_H_26_O_17_	23.91	[M+H]^+^	622.485	623.4117	+	–	[59]
	**Flavanones**								
25	Pinocembrin	C_15_H_12_O_4_	11.31	[M-H]^–^ [M+H]^+^	256.2570	255.1476 257.1037	+	+	[35]
26	Cryptostrobin	C_16_H_14_O_4_	12.46	[M-H]^–^ [M+H]^+^	270.284	269.1739 271.0981	+	+	[32]
27	Strobopinin	C_16_H_14_O_4_	12.72	[M-H]^–^	270.2840	269.1747	–	+	[35]
28	Naringenin	C_15_H_12_O_5_	13.17	[M+H]^+^	272.257	273.2100	+	–	[46]
29	8-Methylpinocembrin	C_16_H_14_O_4_	14.09	[M+H]^+^	270.2840	271.1037	–	+	[35]
	**Flavanones**								
30	7-Hydroxy-5-methoxy-6,8-dimethylflavanone	C_18_H_18_O_4_	15.61	[M-H]^–^	298.3380	297.2050	–	+	[35]
31	8-Prenylnaringenin	C_20_H_20_O_5_	20.45	[M-H]^–^	340.375	339.3448	–	+	[60]
32	6-Geranylnaringenin	C_25_H_28_O_5_	28.56	[M+H]^+^	408.4868	409.4701	–	+	[49]
	**Isoflavone**								
33	Biochanin A	C_16_H_12_O _5_	10.44	[M-H]^–^ [M+H]^+^	284.263	283.1853 285.1370	+	–	[50]
34	4’-*O*-Methylequol	C_16_H_16_O _3_	11.53	[M-H]^–^	256.30	255.1296	–	+	[49]
35	Dihydrodaidzein	C_15_H_12_O_4_	11.63	[M-H]^–^	256.25	255.1142	–	+	[50]
36	Glycitin-6’‘-*O*-acetate	C_24_H_24_O_11_	11.89	[M-H]^–^	488.4	487.4554	–	+	[50]
37	Pseudobaptigenin	C_15_H_10_O_7_	13.17	[M+H]^+^	282.25	283.1075	+	–	[61]
38	Glycitein	C_16_H_12_O _5_	13.96	[M-H]^–^	284.263	283.1666	+	–	[50]
39	Genistein 4’,7-*O* diglucuronide	C_27_H_26_O_17_	28.16	[M-H]^–^	622.485	621.4432	–	+	[62]
	**Chalcones**								
40	Phloretin	C_15_H_14_O_5_	0.66	[M-H]^–^	274.269	273.0029	–	+	[46]
41	6,8-Di-C-methyl pinocembrin-5-methyl ether	C_18_H_18_O _4_	11.67	[M+H]^+^	298.3380	299.1618	+	–	[35]
42	Aurentiacin	C_18_H_18_O _4_	12.06	[M-H]^–^ [M+H]^+^	298.3380	297.1133 299.1569	+	+	[35]
43	Cardamonin	C_16_H_14_O_4_	12.92	[M-H]^–^ [M+H]^+^	270.28	269.1631 271.1143	+	+	[52]
44	Uvangoletin	C_15_H_10_O_6_	13.40	[M-H]^–^	272.3000	271.1406	–	+	[35]
45	2’,4’-Dihydroxy-3’-methyl-6’-methoxy-chalcone	C_17_H_16_O _4_	13.53	[M-H]^–^ [M+H]^+^	284.3065	283.1888 285.2387	+	+	[51]
46	Stercurensin	C_17_H_16_O _4_	13.77	[M-H]^–^ [M+H]^+^	284.3110	283.1625 285.1329	+	+	[35]
47	Demethoxymatteucinol	C_17_H_16_O _4_	14.14	[M-H]^–^ [M+H]^+^	284.3110	283.1846 285.1332	+	+	[35]
48	2’-Hydroxy-4’,6’-dimethoxy chalcone	C_16_H_12_O_5_	14.41	[M-H]^–^	284.307	283.2011	–	+	[49]
49	2’,4’-Dihydroxy-6’-methoxy-3’-methyl dihydrochalcone	C_17_H_18_O_4_	14.65	[M-H]^–^ [M+H]^+^	286.3270	285.2119 287.1356	+	+	[35]
50	2′,4′-Dihydroxy-6′- methoxy3′,5′- dimethyl dihydrochalcone	C_18_H_18_O _4_	15.35	[M-H]^–^ [M+H]^+^	298.3	297.2200 299.1672	+	+	[63]
51	4’,6’-Dihydroxy-3’,5’-dimethyl-2’-methoxy chalcone	C_18_H_18_O_4_	15.45	[M-H]^–^	298.3380	297.1987	–	+	[35]
52	Dimethyl cardamonin	C_18_H_18_O _4_	15.81	[M-H]^–^ [M+H]^+^	298.331	297.2204 299.1618	+	+	[64]
	**Lignin**								
53	Lariciresinol-sesquilignan	C_30_H_36_O_10_	15.82	[M-H]^–^	556.601	555.4398	+	–	[49]
	**Resorcinol derivatives**								
54	5-Heptadecylresorcinol	C_23_H_40_O_2_	7.46	[M-H]^–^ [M+H]^+^	348.56	347.2201 349.2050	+	–	[54]
55	Adipostatin E	C_22_H_38_O_2_	7.67	[M-H]^–^	334.544	333.2106	+	–	[54]
	**Others**								
56	Bergapten	C_12_H_8_O_4_	0.72	[M-H]^–^	216.189	215.1005	–	+	[65]
57	Citric acid	C_6_H_8_O_5_	0.72	[M-H]^–^	192.124	191.0651	–	+	[46]
58	Malic acid	C_4_H_6_O_5_	0.75	[M-H]^–^	134.0874	132.9923	+	+	[46]
59	Quinic acid	C_7_H_12_O_6_	0. 80	[M-H]^–^	192.17	191.1055	+	+	[32]
60	3,5-Dimethyl-resveratrol (Pterostilbene)	C_16_H_16_O_3_	11.79	[M-H]^–^	256.3	255.1323	–	+	[66]
61	Stigmastanol, *trans*-ferulate	C_39_H_60_O_4_	21.93	[M+H]^+^	592.4488	593.3953	+	–	[67]
62	Cyanidin-3-*O*-glucosyl-rutinoside	C_33_H_41_O_2_	31.05	[M+H]^+^	757.2184	758.7891	–	+	[68]

Phenolic acids are widely distributed in all native Australian flora, including *Syzygium* species [43]. Based on their chemical scaffold, they are sub-classified into hydroxybenzoic and hydroxycinnamic acid derivatives [44]. In the current study, we identified three hydroxybenzoic acid derivatives in the negative mode of *S. malaccense* (**1**, **2**, **6**) and five in *S. samarangense* (**1–3**, **5**, **6**). Additionally, one hydroxycinnamic acid derivative (**4**) was detected in the positive mode of *S. malaccense*. Compounds **1**, **2**, **3**, and **5**, were identified as gallic acid, methyl gallate, ellagic acid glucoside, and ellagic acid with m/z [M-H]^-^169.0373, 183.0698, 463.1513, and 301.0638, respectively. Compounds **4** and **6** were identified as hydroxycaffeic acid and gallic acid glucoside with m/z [M + H]^+^ 197.1579 and 333.1248, respectively. Compounds **1, 2**, **5,** and **6** were identified before from *S. samarangense* [32,45], while compound **1** was previously reported from *S. malaccense* [30,46]. The remaining compounds were identified for the first time.

Flavonoids are the most abundant bioactive metabolites widely distributed in medicinal plants. They are crucial metabolites with favorable therapeutic effects on multiple diseases [47]. Chemically, flavonoids comprise 15 carbon atoms in their basic frame, which are allocated as two six-membered rings and one three-carbon unit coupled to them as C6-C3-C6. Based on structural differences, they are classified into seven subclasses: flavonols, flavones, isoflavones, anthocyanidins, flavanones, flavanols, and chalcones [48]. In the current study, we annotated **46** flavonoids, including **15** flavonols, **7** flavanones, **3** flavones, 7 isoflavonoids, and 14 chalcones (Table 2).

Another notable subclass of flavonoids is the flavonols. Herein, eight flavonols **(7, 8**, **10**, **13**, **14**, **17**, **18**, and **19**) were identified from both species. In addition, four (**12**, **15**, **16**, **20**) were identified from *S. malaccense*, and three from *S. samarangense* (**9**, **11**, **21**). Compounds **7** and **8** were identified as myricetin (**7**) and myricetin-*O*-rhamnoside (**8**) with *m/z* [M-H]^-^ 317.1821 and 463.2279, respectively. Compounds **9**, **10**, **11**, **13**, **15**, **16**, **17,** and **18** were identified in the negative mode as isoquercetin (*m/z* [M-H]^-^ 463.1943), quercetin (*m/z* [M-H]^-^ 301.0468, hyperin (*m/z* [M-H]^-^ 463.1249), isorhamnetin-*O*-glucoside (*m/z* [M-H]^-^ 477.1945), quercitrin (*m/z* [M-H]^-^ 447.2192), kaempferol-*O*-glucoside (*m/z* [M-H]^-^ 447.2192), quercetin 4′-O-glucuronide (*m/z* [M-H]^-^ 477.2255), and mearnsitrin (*m/z* [M-H]^-^ 477.2544). On the other side, compounds **12**, **14**, **19, 20** and **21** were identified in the positive mode as isorhamnetin-*O*-rhamnoside (*m/z* [M + H]^+^ 463.1954), mearnsetin (*m/z* [M + H]^+^ 333.11245), kaempferol (*m/z* [M + H]^+^ 287.1558), quercetin 3-*O*-acetyl-galactoside) -*O*-rhamnoside (*m/z* [M + H]^+^ 653.4450) and quercetin 3’-sulfate (*m/z* [M + H]^+^ 383.4042). Compounds **7** (myricetin), **8** (myricetin- *O*-rhamnoside), **10** (quercetin), **14** (mearnsetin), **15** (quercitrin), **18** (mearnsitrin), and **19** (kaempferol) were identified before from *S. malaccense* leaves grown in Brazil but not from the Egyptian species [25,46], while **13 (**isorhamnetin-*O*-glucoside), **16** (kaempferol-*O*-glucoside were identified from its fruits [46]. Flavonols **12** (isorhamnetin-*O*-rhamnoside), **17** (quercetin 4′-*O*-glucuronide), and **20** (quercetin 3-*O*-acetyl-galactoside)-*O*-rhamnoside) were detected for the first time. Ultimately, **compounds 8** (myricetin-*O*-rhamnoside), **9** (isoquercitrin), **10** (quercetin), and **11** (hyperin) were identified before from the leaves of *S. samarangense* grown in Egypt [32,45], compounds **8**, **10**, and **11** were identified from its fruits, while compounds **7** and **19** were detected in its stem bark [36]. Flavonols **7**, **13**, **14**, **17**, **18**, **19**, and **21** were tentatively identified for the first time from *S. samarangense* leaves. Additionally, two flavone glycosides were identified in the positive mode of *S. malaccense* as diosmin (**23**) (*m/z* [M + H]^+^ 609.4222) and apigenin-7-*O*-diglucuronide (**24**) (*m/z* [M + H]^+^ 623.4117), while 4’,7-tetrahydroxyflavone (**22**) (*m/z* [M-H]^-^ 285.1601) was identified in the negative mode of *S. samarangense*. This is the first time to identify the forementioned flavone glycosides from both species, though **22** and **23** were previously detected in other *Syzygium* species [49]. Concerning the flavanone sub-class, eight of them were identified in the analyzed extracts. Pinocembrin **25** (*m/z* [M-H]^-^ 255.1476/ [M + H]^+^ 257.1037) and cryptostrobin **26** (*m/z* [M-H]^-^ 269.1739/ [M + H]^+^ 271.0981) were identified in both negative and positive modes of the two species. Naringenin **28** was identified in the positive mode of *S. malaccense* (*m/z*[M + H]^+^ 273.2100) while strobopinin **27** (*m/z* [M-H]^-^ 269.1747), 7-hydroxy-5-methoxy-6,8-dimethylflavanone **30** (m/z [M-H]^-^ 297.2050), and 8-prenylnaringenin **31** (m/z [M-H]^-^ 339.3448) were identified in the negative mode of *S. samarangense.* Additionally, 8-methylpinocembrin **29** (m/z [M + H]^+^ 271.1037) and 6-geranyl naringenin **32** (*m/z*[M + H]^+^ 409.4701) were identified in its positive mode. This is the first time identifying the flavanones class of metabolites in *S. malaccense,* except naringenin **28,** which was identified before in the Brazilian species [46]. **26** was previously detected in the leaf extract of *S. samarangense* cultivated in Egypt [32], while **25**, **27**, **29**, and **30** were identified from its stem bark [35]. **31** and **32** were identified for the first time from the Egyptian species, while previously reported in other Myrtle species [49]. Skimming the presence of other flavonoid subclasses, three isoflavones were tentatively identified in *S. malaccense.* One of which, namely biochanin A **33** (*m/z* [M-H]^-^ 283.1853/ [M + H]^+^ 285.1370), is detected in both negative and positive modes. On the other hand, **37** and **38** were identified as pseudobaptigenin (*m/z* [M + H]^+^ 269.1739) and glycitein *m/z* [M-H]^-^ 283.1666), respectively. Moreover, 4’-*O*-methyl equol **33**, dihydrodaidzein **35**, glycitin-6’‘-*O*-acetate **36,** and genistein 4’,7-*O*-diglucuronide **39** were identified in the negative mode of *S. samarangense* with *m/z* [M-H]^-^ 255.1296, 255.1142, 487.4554, and 621.4432, respectively. Interestingly, this is the first report of isoflavones from the two species investigated, but they have been previously detected in other Myrtaceae species [49,50].

Chalcones and their derivatives represent an interesting category of flavonoids due to their versatility and effective bioactivities. They comprise two aromatic rings bridged by an α, β-unsaturated system of three carbons. In this regard, nine **(41–43, 45–47, 49, 50,** and **52)** and twelve **(40, 42–52)** chalcones were identified from *S. malaccense and S. samarangense*, respectively. Chalcones **42**, **43**, **45**, **46**, **47**, **49**, **50**, and **52** were detected in positive and negative modes of both species and elucidated as aurentiacin **42** (*m/z* [M-H]^-^ 297.1133/ [M + H]^+^ 299.1569), cardamonin **43** (*m/z* [M-H]^-^ 269.1631/ [M + H]^+^ 271.1143), 2’,4’-dihydroxy-6’-methoxy-3’-methyl chalcone **45** (*m/z* [M-H]^-^ 283.1888/ [M + H]^+^ 285.2387), stercurensin **46** (*m/z* [M-H]^-^ 283.1625/ [M + H]^+^ 285.1329), demethoxymatteucinol **47** (*m/z* [M-H]^-^ 283.1846/ [M + H]^+^ 285.1332), 2’,4’-dihydroxy-6’-methoxy-3’-methyl dihydrochalcone **49** (*m/z* [M-H]^-^ 285.2119/ [M + H]^+^ 287.1356), 2′,4′-dihydroxy-6′- methoxy3′,5′- dimethyl dihydrochalcone **50** (*m/z* [M-H]^-^ 297.2200/ [M + H]^+^ 299.1672) and dimethyl cardamonin **52** (*m/z* [M-H]^-^ 297.2204/ [M + H]^+^ 299.1618). On the other side, 6,8-di-*C*-methyl pinocembrin-5-methyl ether **41** was detected in the positive mode of *S.malaccense* (*m/z* [M + H]^+^ 299.1618), while phloretin **40**, uvangoletin **44**, 2’-hydroxy-4’,6’-dimethoxy chalcone **48** and 4’,6’-dihydroxy-3’,5’-dimethyl-2’-methoxy chalcone **51** were detected in the negative mode of *S. samarangense* with *m/z* [M-H]^-^ 273.0029, 271.1406, 283.2011 and 297.1987, respectively. Interestingly, this is the first study to report the detection of chalcones in the leaves of *S. malaccense*, while chalcones have been reported before from *S. samarangense.* For instance, **45** was previously identified in *S. samarangense* leaves’ extract [51], compounds **42**, **44**, **46**, **47**, **49**, **51,** and **52** were detected in its stem bark [35], and compound **43** from the fruits [52] as well as from other *Syzigium* species [49,51].

Lignin is the most vital and profuse biopolymer that connects cellulose and hemicellulose fibers, providing a rigorous structure to the plant cell [53]. Herein, only one lignin, lariciresinol-sesquilignan **53,** was identified for the first time from the negative mode of *S. malaccense* with (*m/z* [M-H]^-^ 555.4398), but it was previously detected from other Myrtaceae species [49]. Two derivatives were identified for the first time from *S. malaccense*, one of which is 5-heptadecylresorcinol **54** and detected in both ionization modes (*m/z* [M-H]^-^ 347.2201/ [M + H]^+^349.2050), while the other is adipostatin E **55** and detected in the negative mode only (*m/z* [M-H]^-^ 255.1296). The two compounds were identified earlier from the leaves of *S. samarangense* grown in China [54].

Ultimately, three miscellaneous metabolites were detected for the first time in *S. malaccense* extract and elucidated as malic acid **58** (*m/z* [M-H]^-^ 132.9923), quinic acid **59** (*m/z* [M-H]^-^ 191.1055), and stigmastanol *trans*-ferulate **61 (***m/z* [M + H]^+^ 593.3953). To our knowledge, compounds **58** and **59** were previously identified from *S. samarangense* [32,46]. On the other side, bergapten **56** (*m/z* [M-H]^-^ 215.1005), citric acid **57 (***m/z* [M-H]^-^ 191.0651), 3,5-dimethyl-resveratrol **60** (*m/z* [M-H]^-^ 255.1323), cyanidin 3-*O*-glucosyl-rutinoside **62 (***m/z* [M + H]^+^ 758.7891).

### Acute toxicity study

The acute toxicity study was performed to ensure the safe dosing range of the extracts before proceeding to further pharmacological evaluations. Understanding the acute toxicity profile allows the selection of appropriate doses for efficacy studies while minimizing potential adverse effects. Herein, twenty-four hours post-extract administration, no changes were observed in the motor activity or the behavior of the tested animals, and no mortality was recorded. Therefore, both DEs were considered safe till 5 g/kg, and, consequently, we selected dose levels of 250, 500, and 1000 mg/kg to evaluate the extract’s nephroprotective effect.

### *In vivo* nephroprotective activity

#### Effect of the DE of *S. malaccense* and *S. samarangense* on the levels of urea and creatinine.

As seen in Table 3, CdCl_2_ induced kidney tissue damage that affected the function of the glomeruli, evidenced by the significant (*p* < 0.001) increase in the levels of urea and creatinine in CdCl_2_-treated rats by 4.4-fold and 3.7-fold, respectively, compared to the control group. Both tested extracts preserved the renal function evidenced by the significant (*p* < 0.001) decrease in urea and creatinine levels as follows: DE of *S. malaccense* in doses 250, 500, and 1000 mg/kg significantly declined urea levels by 33.5%, 64.1%, and 69.2%, respectively; and decreased creatinine levels by 22.3%, 52.9%, and 60.6%, respectively, compared to the CdCl_2_-treated rats. Meanwhile, *S. samarangense* significantly (*p* < 0.001) decreased urea levels by 45.3%, 68.9%, and 70.2%, respectively, and decreased creatinine levels by 25.8%, 61.5%, and 65.4%, respectively, compared to the CdCl_2_-treated rats.

**Table 3:** Effect of different doses of the defatted extract (DE) of the leaves of *S. malaccense* and *S. samarangense* on urea and creatinine levels. Data represented as mean±SEM of n = 7, a: significant from the control group at *p* < 0.001, b: significant from the CdCl_2_ group at *p* < 0.001.

	Control	CdCl_2_	*S. malaccense*			*S. samarangense*		
			250	500	1000	250	500	1000
**Urea (µmol/mL)**	6.674 ± 0.33	29.06 ± 0.61 ^a^	19.33 ± 0.74 ^a,b^	10.44 ± 0.27 ^a,b^	8.952 ± 0.13 ^a,b^	15.89 ± 0.48 ^a,b^	9.017 ± 0.78 ^a,b^	8.648 ± 0.31 ^a,b^
**Creatinine** **(nmol/mL)**	0.4433 ± 0.02	1.630 ± 0.01 ^a^	1.266 ± 0.02 ^a,b^	0.768 ± 0.03 ^a,b^	0.641 ± 0.03 ^a,b^	1.209 ± 0.03 ^a,b^	0.628 ± 0.01 ^a,b^	0.563 ± 0.02 ^a,b^

#### Effect of the DE of *S. malaccense* and *S. samarangense* leaves on oxidative stress parameters.

**Fig 3:**
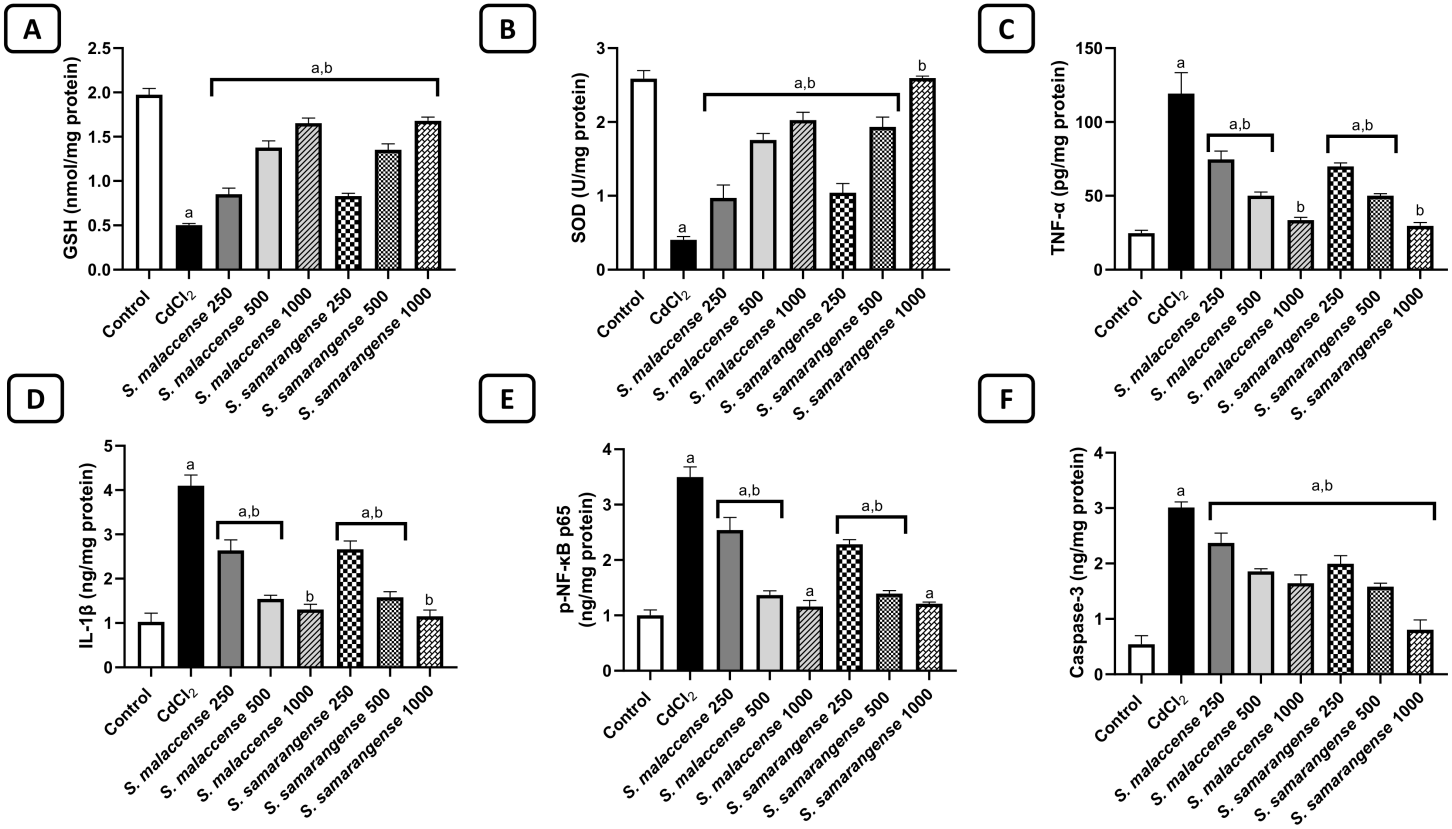
Effect of DE of *S. malaccense* and *S. samarangense* on A) GSH, B) SOD, C) TNF-α, D) IL-1β, E) p-NF-κB, and F) Caspase-3 levels. Data represented as mean±SEM of n = 7. a: significant from the control group at **p* *< 0.001, b: significant from the CdCl_2_ group at *p* < 0.001.

Oxidative damage induced by CdCl_2_ is evidenced by the marked (*p* < 0.001) decrease in GSH by 74.6% and SOD by 84.4%, compared to the control group. Doses of 250, 500, and 1000 mg/kg from the DE of *S. malaccense* significantly (*p* < 0.001) boosted the level of GSH by 1.7, 2.7, and 3.3 folds, respectively; and SOD by 2.4, 4.3, and 5 folds, respectively, compared to CdCl_2_-treated group. Meanwhile, doses of 250, 500, and 1000 mg/kg from *S. samarangense* significantly (*p* < 0.001) enhanced the level of GSH by 1.7, 2.7, and 3.3 folds, respectively. In addition, they potentiated the level of SOD by 2.6, 4.8, and 6.4 folds, respectively, compared to the CdCl_2_-treated group. These findings support their antioxidant property (Figs 3A and 3B).

#### Effect of the DE of *S. malaccense* and *S. samarangense* leaves on inflammatory parameters.

Inflammation is involved in CdCl_2_-induced damage, as seen in CdCl_2_-treated rats. They manifested a marked (*p* < 0.001) elevation in TNF-*α*, IL-1*β*, and p-NF-κB p65, by 4.8, 4.0, and 3.5 folds, respectively, compared to the control rats. Treatment with *S. malaccense* DE (250, 500, and 1000 mg/kg) significantly (*p* < 0.001) reduced TNF-*α* by 37.4%, 57.9%, and 71.9%; decreased IL-1*β* by 35.6%, 62.4%, and 68.1%; and reduced p-NF-κB p65 by 27.3%, 60.9%, and 66.8%, respectively, compared to the CdCl_2_-treated rats. Additionally, treatment with *S. samarangense* (250, 500, and 1000 mg/kg) significantly (*p* < 0.001) decreased TNF-*α* by 41.3%, 57.9%, and 75.1%; and reduced IL-1*β* by 34.9%, 61.4%, and 71.9%; and decreased p-NF-κB p65 by 34.7.3%, 60.1%, and 65.4%, respectively, compared to the CdCl_2_-treated rats. These results indicate the promising anti-inflammatory activity of the tested extracts (Figs 3C-3E).

#### Effect of the DE of *S. malaccense* and *S. samarangense* leaves on the apoptotic parameter caspase-3.

Treatment with CdCl_2_ induced apoptosis of renal tissue as indicated by the marked (*p* < 0.001) increase in caspase-3 level in CdCl_2_-treated rats by 5.6-fold compared to the control rats. Meanwhile, administration of *S. malaccense* (250, 500, and 1000 mg/kg) significantly (*p* < 0.001) reduced the caspase-3 level by 21.2%, 38.2%, and 45.4%, respectively, compared to the CdCl_2_-treated rats. Moreover, treatment with *S. samarangense* (250, 500, and 1000 mg/kg) significantly (*p* < 0.001) decreased the caspase-3 level by 33.8%, 47.5%, and 73.3%, respectively, compared to the CdCl_2_-treated rats. These results suggest the anti-apoptotic and cytoprotective effect of *S. malaccense* and *S. samarangense* DEs (Fig 3F).

#### Effect of the DE of *S. malaccense* and *S. samarangense* leaves on the mitochondrial dysfunction parameters.

**Fig 4:**
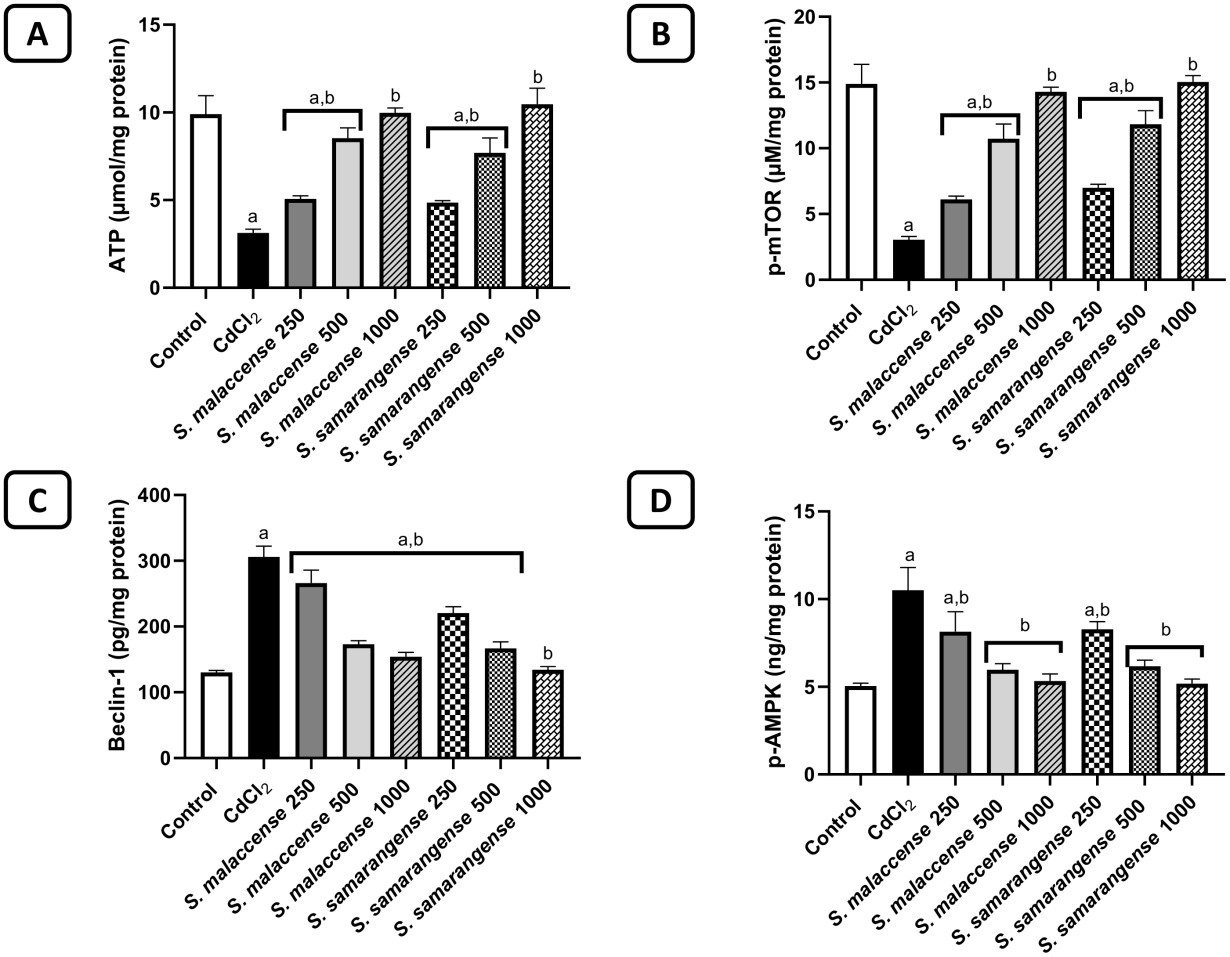
Effect of DE of *S. malaccense* and *S. samarangense* leaves on A) ATP, B) p-mTOR, C) beclin-1, and D) p-AMPK levels. Data represented as mean±SEM of n = 7. a: significant from the control group at **p* *< 0.001, b: significant from the CdCl_2_ group at *p* < 0.001.

The CdCl_2_-treated group showed a significant (*p* < 0.001) reduction in ATP by 68.5% compared to the control rats. Groups treated with the DE of *S. malaccense* at doses 250, 500, and 1000 mg/kg showed a marked (*p* < 0.001) increase in ATP by 1.6, 2.7, and 3.2 folds, respectively, compared to the CdCl_2_-treated group. Additionally, groups administered the DE of *S. samarangense* at doses 250, 500, and 1000 mg/kg showed a significant (*p* < 0.001) increase in ATP by 1.6, 2.5, and 3.3 folds, respectively, compared to the CdCl_2_-treated rats (Fig 4A).

#### Effect of the DE of *S. malaccense* and *S. samarangense* leaves on autophagy parameters.

Induction of autophagy was detected by measuring *p*-mTOR and beclin-1. As presented in Figs 4B and 4C, the CdCl_2_-treated group demonstrated a marked (*p* < 0.05) decrease in *p*-mTOR by 79.6% and a marked (*p* < 0.001) increase in beclin-1 by 2.4-fold compared to the control group. Meanwhile, groups administered with 250, 500, and 1000 mg/kg of *S. malaccense* DE significantly (*p* < 0.001) increased *p*-mTOR by 2, 3.5, and 4.7 folds; and decreased beclin-1 by 12.9%, 43.3%, and 49.5%, respectively, compared to CdCl_2_-treated groups. Groups treated with 250, 500, and 1000 mg/kg of *S. samarangense* DE significantly (*p* < 0.001) increased *p*-mTOR by 2.3, 3.9, and 4.9 folds; and decreased beclin-1 by 27.8%, 45.4%, and 56.1%, respectively, compared to CdCl_2_-treated groups. Regarding the level of p-AMPK, the CdCl_2_-treated group showed a marked (*p* < 0.001) elevation in p-AMPK by 2.1-fold compared to the control group. Groups treated with the DE of *S. malaccense* at doses 250, 500, and 1000 mg/kg showed a significant (*p* < 0.001) decrease in p-AMPK by 22.4%, 43.2%, and 49.2%, respectively, compared to the CdCl_2_-treated group. Additionally, groups administered the DE of *S. samarangense* at doses 250, 500, and 1000 mg/kg showed a marked (*p* < 0.001) decrease in p-AMPK by 21.1%, 41.2%, and 50.6%, respectively, compared to the CdCl_2_-treated group (Fig 4D).

#### Effect of the DE of *S. malaccense* and *S. samarangense* on serum ALT and AST levels.

As delineated in Table 4, CdCl_2_-treated rats exhibited a significant elevation in serum ALT and AST levels compared to the control group (p < 0.001) by 2.8 and 2.9 folds, respectively, indicating systemic hepatic toxicity. Treatment with the DE of *S. malaccense* at doses 250, 500, and 1000 mg/kg significantly decreased the serum level of ALT by 26.1%, 33.3%, and 57.5%, and AST by 34.9%, 53.4%, and 58.6%, respectively, compared to the CdCl_2_ group (p < 0.05). Meanwhile, treatment with the DE of *S. samarangense* at 250, 500, and 1000 mg/kg significantly decreased the serum level of ALT by 30.5%, 46.8%, and 58.6%, and AST by 35.4%, 56.1%, and 62.0%, respectively, compared to the CdCl_2_ group (p < 0.05). These results suggest a protective effect of the DEs against CdCl2-induced liver injury.

**Table 4:** Effect of different doses (mg/Kg) of the defatted extract (DE) of the leaves of *S. malaccense* and *S. samarangense* on serum ALT and AST levels. Data represented as mean±SEM of n = 7, a: significant from the control group at *p* < 0.001, b: significant from the CdCl_2_ group at *p* < 0.001.

	Control	CdCl_2_	*S. malaccense* (mg/Kg)			*S. samarangense* (mg/Kg)		
			250	500	1000	250	500	1000
**ALT (ng/mL)**	1.41 ± 0.04	4.08 ± 0.16 ^a^	3.02 ± 0.11 ^a,b^	2.72± 0.09 ^a,b^	1.73± 0.06 ^a,b^	2.84± 0.08 ^a,b^	2.17± 0.07 ^a,b^	1.69 ± 0.06 ^a,b^
**AST** **(ng/mL)**	1.89 ± 0.06	5.55 ± 0.29 ^a^	3.61 ± 0.10 ^a,b^	2.58± 0.11 ^a,b^	2.29± 0.05 ^a,b^	3.58± 0.14 ^a,b^	2.43± 0.12 ^a,b^	2.10 ± 0.06 ^a,b^

#### Effect of the DE of *S. malaccense* and *S. samarangense* leaves on histopathological examination of the kidney tissues.

**Fig 5:**
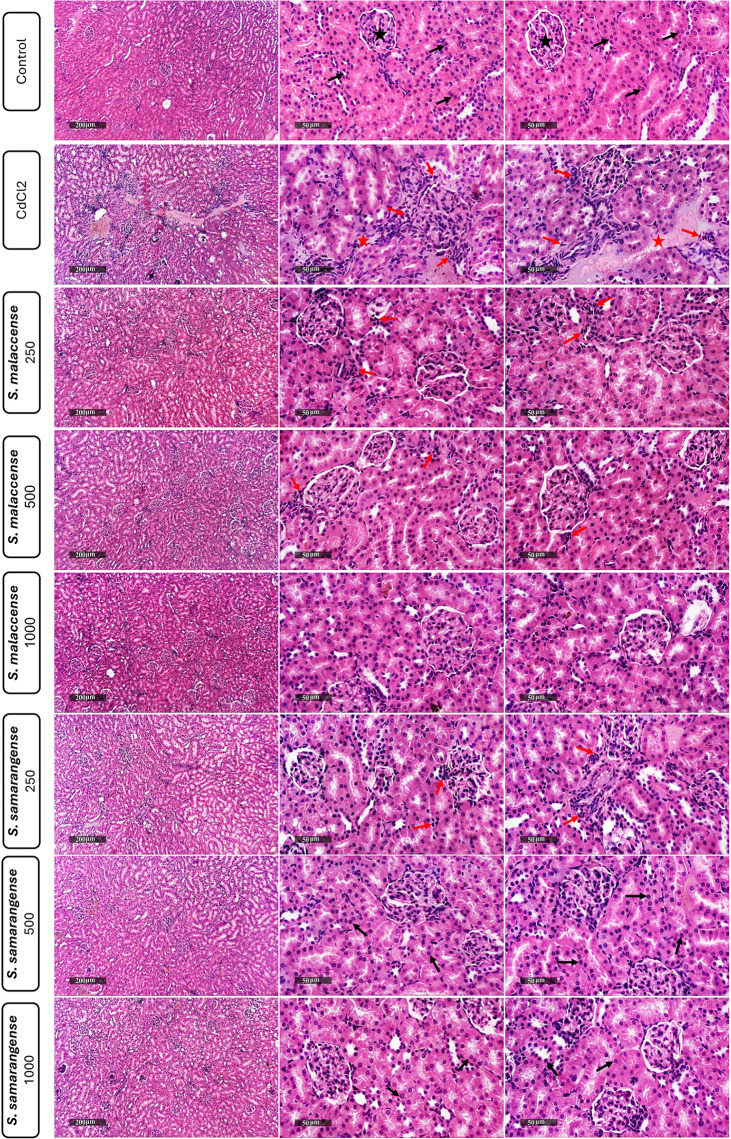
Effect of DE of *S. malaccense* and *S. samarangense* leaves on histopathological examination of the kidney tissues. Normal kidney samples showed intact renal parenchyma with renal tubular segments and intact tubular epithelium (arrow), intact renal corpuscles (star), and intact vasculature. CdCl_2_-treated samples showed multiple focal periglomerular and perivascular mononuclear inflammatory cell infiltrates accompanied by higher fibroblastic activity (red arrow) with dilatation of renal vasculatures (red star). DEs of *S. malaccense* and *S. samarangense* at 250 mg/kg doses showed mild perivascular and periglomerular inflammatory cell infiltrates (red arrow) with almost intact vasculatures and nephron segments. *S. malaccense* at a dose of 500 mg/kg showed higher protective efficacy with few occasional interstitial inflammatory cell infiltrates (red arrow). In comparison, the 1000 mg/kg showed higher protective efficacy with almost intact renal parenchyma. 500 mg/kg and 1000 mg/kg of *S. samarangense* showed a higher protective efficacy with almost intact renal parenchyma resembling normal control samples.

As seen in Fig 5, normal kidney samples showed almost intact, well-organized morphological features of renal parenchyma. It showed apparent intact renal tubular segments and intact tubular epithelium (arrow), minimal records of degenerated tubular epithelial cells, intact renal corpuscles (star), and intact vasculatures. However, CdCl_2_-treated samples showed multiple focal records of periglomerular and perivascular mononuclear inflammatory cell infiltrates accompanied by higher fibroblastic activity (red arrow) with dilatation of renal vasculatures (red star). Both DEs of *S. malaccense* and *S. samarangense* (250 mg/kg) showed mild persistent records of perivascular and periglomerular inflammatory cell infiltrates (red arrow) with almost intact vasculatures and apparent intact nephronal segments. *S. malaccense* at a dose of 500 mg/kg showed higher protective efficacy with a few occasional records of interstitial inflammatory cell infiltrates (red arrow). In comparison, 1000 mg/kg demonstrated higher protective efficacy with almost intact renal parenchyma resembling normal control samples. The 500 mg/kg and 1000 mg/kg of *S. samarangense* showed higher protective efficacy with almost intact renal parenchyma resembling normal control samples.

## Discussion

The kidneys are among the most energy-intensive organs, crucial for maintaining water and salt equilibrium [69]. They receive nearly 25% of the cardiac output and are responsible for blood pressure management and continuous blood filtration [70]. Cadmium (Cd) pollution is escalating globally due to heightened industrial operations, which have augmented its availability and environmental persistence. Furthermore, it poses a significant public health hazard owing to its non-biodegradability and extended biological half-life (10–30 years) [71]. Environmental Cd may accumulate in different human organs, including the liver and lungs. However, the kidney is still the main target of its accumulation [72,73], making it mainly susceptible to Cd-mediated nephrotoxicity. Flavonoids are unique, natural bioactive secondary metabolites with a basic flavan skeleton. They comprise two phenyl rings connected to a central heterocyclic pyran scaffold, forming a C6-C3-C6 system with a distinguished polyphenolic feature. Flavonoids are allocated into six major groups, *viz.* flavanols, flavanones, flavonols, flavones, isoflavonoids, and anthocyanidins, in addition to other minor classes including chalcones, dihydrochalcones, and aurones. Flavonoids are found extensively in plant-based meals, so they are ingested through fruits and vegetables [74]. They exhibited beneficial pharmacological effects, including antioxidant, free radical scavenging, anti-inflammatory, immunomodulatory, and renal protective effects [75]. Given the global health burden of Cd toxicity on human health, and the potential therapeutic impacts of polyphenols, especially flavonoids, we investigated the protective potential of two phenolic-rich Syzygium species against cadmium chloride-induced nephrotoxicity in rats. The study showed high serum creatinine and urea levels in Cd-treated rats, indicating kidney injury. The levels of these parameters are usually used as biomarkers for assessing kidney function [76]. According to Gowda et al. [77], urea is a byproduct of the breakdown of proteins and amino acids, and an increase in its blood level is typically linked to renal failure or illness. Another biomarker is creatinine, which is mainly removed by glomerular filtration and created during muscle metabolism from creatine and phosphocreatine. In the current investigation, we reported that the DE of *S. malaccense* and *S*. *samarangense* reversed Cd-induced nephrotoxicity, evidenced by decreased blood creatinine and urea levels. This finding follows previous reports, which revealed that phenolic compounds improved the nephrotoxicity induced by heavy metals such as Cd [78–80].

Oxidative damage accompanied by free radical overproduction is one of the main processes that are activated upon Cd intoxication. It was established that Cd causes oxidative stress by influencing the pro-to-antioxidant ratio. Additionally, in the biological system, Cd does not undergo redox processes, however, it can generate oxidative stress by intracellular GSH reduction [81], since Cd can bind with the functional thiol (-SH) groups in both enzymatic and non-enzymatic cellular compounds [82]. Moreover, Cd can inhibit other antioxidant enzymes such as SOD which was attributed to the Cd interaction with the enzyme protein part causing a configurational change in the enzyme and results in depressing SOD catalytic activity [83]. The two examined extracts are rich mainly in flavonoids belonging to various classes, exemplified in flavonols **(7−21),** flavone **(22−24),** flavanones **(25–32)**, isoflavones **(33−39)**, and chalcones **(40−52)**. Flavonoids exert their antioxidant effect *via* direct and indirect mechanisms. Directly by donating electrons that neutralize reactive oxygen species (ROS) [84] and free radicals such as peroxynitrite (ONOO^−^), hydroxyl radical (OH·), and peroxyl radical (ROO·), hence decreasing ROS and other free radicals levels in the body [85]. Though the sugar part of the flavonoid skeleton is critical for their antioxidant activity, the aglycones are more effective than their corresponding glycosides [86]. On the other hand, the indirect antioxidant effect of flavonoids is associated with inducing the production or activation of antioxidant enzymes and suppressing pro-oxidant enzymes. Flavonoids can activate the intracellular antioxidant signaling pathways to induce the production of intracellular antioxidant elements including GSH and SOD. We cannot neglect the effect of other phenolic classes detected in the two tested DE, such as phenolic acids **(1−6),** resveratrol derivatives (**60**), and lignans (**53**), which are all characterized by their potential antioxidant effect [49,87]. In all, our result revealed that the reduced GSH and SOD levels in the Cd-administrated rats were significantly restored by using the DE of *S. malaccense* and *S. samarangense,* especially at higher doses (500 and 1000 mg/kg). Moreover, several flavonoids and phenolic acids identified in this study were previously reported to have a strong antioxidant effect [33,47,49] and protect against kidney injury [75,87,88]. It is well-known that oxidative stress and inflammation are interrelated processes where elevated levels of ROS enhance NF-κB activation and release of inflammatory cytokines. NF-κB is an essential modulator of the immune system and several inflammatory disorders. Furthermore, it is essential to activate inflammatory cytokines including IL-1β and TNF-α [89]. Prior research has demonstrated that exposure to Cd in renal tissues can trigger the activation of NF-κB, leading to an increase in TNF-*α*, IL-1, and IL-6 [82,90,91]. Our study demonstrated that the examined extracts significantly decreased the levels of NF-κB, TNF-*α*, and IL-1*β*, especially at higher doses. These results might be attributed to flavonoids, confirmed by earlier reports that disclosed the anti-inflammatory potential of flavonoids and phenolic acids [33,47,49]. Through a variety of processes, including immune cell regulation and enzyme and transcription factor inhibition, flavonoids produce their anti-inflammatory effects. Prior research has demonstrated that flavonoids affect immune cell development, activation, and signaling transduction, which can interfere with cytokine and chemokine synthesis and secretion [47].

Cd-induced oxidative stress and inflammation may trigger apoptosis which has an essential role in Cd-caused nephrotoxicity [92]. Apoptosis results from an imbalance between apoptotic and anti-apoptotic molecules, which is caused by both dependent and independent mitochondrial pathways [5]. Because it can initiate the apoptotic pathway by inducing other caspase enzymes, caspase-3 is considered an essential apoptotic marker [93]. A previous report coincides with our finding that Cd exposure significantly elevates caspase-3 level in the rat’s renal tissues [6,94]. In the present study, treatment with our investigated extracts protected against Cd-induced renal apoptosis by inhibiting the activation of caspase-3, which indicates their anti-apoptotic potential against Cd exposure. This effect may be at least in part, due to the extracts’ flavonoid and phenolic acids content. Both are renowned for their valuable therapeutic activity on renal health due to their antioxidant, anti-inflammatory, and anti-apoptotic properties [75].

Mitochondria are referred to as the engine of the cell due to their capability to generate ATP via oxidative phosphorylation [95]. They are critical organelles in preserving cellular homeostasis, and therefore, their damage may result in tissue or cellular damage [96]. Renal mitochondrial dysfunction is associated with inflammation, apoptosis, and tissue damage, which raises the risk of death and morbidity [97]. It was documented that Cd exposure decreases the level of ATP because of mitochondrial damage [98], which is consistent with our result. Meanwhile, the level of ATP is increased upon the treatments with the two extracts investigated.

Autophagy is an evolutionary conserved catabolic process used by eukaryotic cells to break down damaged or unnecessary proteins and organelles [99]. It is crucial for the upkeep and survival of cells [100]. It participates in Cd-induced nephrotoxicity [101]. Furthermore, it has been reported to be the early response of a cell to Cd toxicity in a concentration- and time-dependent way [102]. During the early stage of exposure, low levels of Cd may trigger protective autophagy in renal tubular epithelial cells [103–105], but with continuous exposure, excessive autophagy ultimately fails to maintain cell viability and then triggers cell death [7]. Autophagy can result from the activation of many signaling pathways by cellular proteins and/or protein kinases [106]. Amongst them, the AMPK/mTOR is the most significant pathway in autophagy regulation [107]. AMPK usually motivates autophagy by impairing mTOR, which plays an important role in monitoring cell growth, proliferation, and autophagy [108]. Moreover, beclin-1 controls autophagy membrane biosynthesis as it is necessary to initiate autophagosome formation [109]. It was reported that Cd exposure significantly decreases mTOR levels, activates the AMPK, and increases protein levels of beclin-1 [110], which is consistent with our results. Meanwhile, our results showed that the administration of both DEs significantly suppressed the excessive autophagy process, evidenced by decreased levels of AMPK and beclin-1 and increased levels of mTOR. The suppressed autophagy effect of the extracts can be elucidated by their antioxidant, anti-inflammatory, antiapoptotic, and cytoprotective activities.

Histopathological analysis of the kidney tissues showed that Cd caused numerous pathological lesions characterized by inflammatory cell infiltration and vascular dilatation. Additionally, the usual histological features of the renal tubules were lost. Cd exposure results in multiple focal records of periglomerular and perivascular mononuclear inflammatory cell infiltrates accompanied by higher fibroblastic activity with dilatation of renal vasculatures. These histomorphological alterations may be due to the direct damage of the renal tissues and increased ROS generation brought on by exposure to Cd [111]. Hence, resulted in oxidative damage [112] and morphological changes in renal tissue. However, the *S. malaccense* and *S. samarangense* DEs, especially at higher doses, display potential protective effects on the kidney tissues, which may be related to the strong antioxidant potential of flavonoids. Flavonoids significantly attenuated the oxidative stress, causing a decrease in pathological changes [49,87].

Cd is well-documented to induce not only nephrotoxicity but also systemic toxicity, particularly hepatotoxicity, through oxidative stress and mitochondrial dysfunction [113,114]. Elevated ALT and AST levels have been observed in the CdCl_2_ group, confirming hepatic injury. The ability of the DE of *S. malaccense* and *S. samarangense* to reduce these liver enzyme levels suggests a broader systemic protective role, possibly *via* antioxidant or anti-inflammatory effects.

## Conclusion

The current research reported that the defatted aqueous methanol extracts (DE) of *S. malaccense* and *S. samarangense* leaves are rich in various classes of flavonoids, such as flavonols, flavone, flavanones, isoflavones, and chalcones, in addition to other phenolic compounds. The administration of the two investigated extracts significantly attenuated Cd-induced renal toxicity by fighting renal oxidative stress, apoptosis, and inflammatory reactions. Moreover, the two extracts alleviated mitochondrial dysfunction and inhibited the autophagy level in a dose-dependent manner. The nephroprotective effects of the two *Syzygium* extracts against Cd-induced nephrotoxicity may be attributed to anti-inflammatory, antiapoptotic, and antioxidant activities of their constitutive phenolic metabolites. Moreover, the DE not only protects renal function but also mitigates systemic Cd-induced hepatic toxicity. However, implementing *in vitro* experiments using renal cell lines and setting quality control parameters for the HPLC-MS are among the study limitations. In all, the two *Syzygium* extracts can be used, at least in part, as a protective agent against environmental toxicity by heavy metals such as cadmium. Nevertheless, future clinical studies are required to test their toxicity and curing ability for Cd-induced renal dysfunctions in humans. Furthermore, formulating these extracts into a suitable pharmaceutical preparation will facilitate their usage as protective nutraceuticals for kidney nephrotoxicity.

### Studies in animal statements

The Faculty of Pharmacy’s Ethical Animal Care and Use Committee approved the experimental protocol at Helwan University (approval number: 11A2023). The animal experiment complied with ARRIVE (Animal Research: Reporting of *In Vivo* Experiments) guidelines. Also, it followed the guidance on the operation of the Animals (Scientific Procedures) Act 1986, the association guidelines, EU directive 2010/63 for the protection of animals used for scientific purposes, the NIH (National Research Council) Guide for the Care and Use of Laboratory Animals (8^th^ edition), and national guidelines for animal care (European Community Directive, 6/609/EEC).

## Supporting information

S1 Checklist Humane endpoints checklist. This checklist details the implementation of humane endpoints in the animal experiment described in the study. It includes information on monitoring criteria, euthanasia timing, animal welfare considerations, and compliance with ethical standards, as per PLOS ONE and ARRIVE guidelines.
(DOCX)
