# Supplementary material for: HPLC-MS profiling and protective potential of the defatted aqueous methanol extract of two Syzygium species against cadmium chloride-induced nephrotoxicity in rats
Source: PLoS One. 2025 Aug 14;20(8):e0329586. doi: 10.1371/journal.pone.0329586 (PMC12352781; doi:10.1371/journal.pone.0329586)
Supplement: S1 Checklist — This checklist details the implementation of humane endpoints in the animal experiment described in the study. It includes information on monitoring criteria, euthanasia timing, animal welfare considerations, and compliance with ethical standards, as per PLOS ONE and ARRIVE guidelines. (DOCX) [file pone.0329586.s001.docx]

*PLOS ONE* Humane Endpoints Checklist

*PLOS ONE* manuscript number: _______________________

**Complete the following if your study design includes death of a regulated animal as a likely outcome or planned experimental endpoint. Please also include all information in the Methods section of your manuscript.**

**ITEM 1.** **Describe whether humane endpoints* were used for all animals involved in the study.**

|  | **Recommendation** | **Section/Paragraph** |
| --- | --- | --- |
| **If humane endpoints* were used, report the following:** | | |
| **1** | **The specific criteria used to determine when animals should be euthanized** | **Methods section – Paragraph on animal monitoring and humane endpoints**  **Lines 276-287** |
| **2** | **Once animals reached endpoint criteria, the amount of time elapsed before euthanasia** | **Methods section – Immediate euthanasia was performed upon meeting endpoint criteria**  **Lines 283-284** |
| **3** | **Whether any animals died before meeting criteria for euthanasia** | **Methods section – No animals died unexpectedly or before meeting humane endpoints**  **Line 284-285** |
| **If humane endpoints* were not used, report the following:** | | |
| **1** | **A scientific and ethical justification for the study design, including the reasons why humane endpoints could not be used, and discussion of alternatives that were considered but could not be used** | **N/A for our study** |
| **2** | **Whether the institutional animal ethics committee specifically reviewed and approved the anticipated mortality in the study design** | **N/A for our study** |

**ITEM 2.** **Include the following details of the study design and outcomes.**

|  | **Recommendation** | **Section/Paragraph** |
| --- | --- | --- |
| **1** | **The duration of the experiment** | **Methods section – The duration of the experiment was 7 days**  **Line 298** |
| **2** | **The numbers of animals used, euthanized, and found dead (if any); the cause of death for all animals** | **Methods section – Total number of animals (56 rats), euthanasia according to humane endpoints; no animals found dead unexpectedly.**  **Line 297** |
| **3** | **How frequently animal health and behavior were monitored** | **Methods section – Animals were monitored at least twice daily**  **Line 278-279** |
| **4** | **All animal welfare considerations taken, including efforts to minimize suffering and distress, use of analgesics or anaesthetics, or special housing conditions** | **Methods section – the details on housing, temperature, humane endpoints, and euthanasia protocol were mentioned in the paragraph from lines 267-287** |
| **5** | **Any special training in animal care or handling provided for research staff** | **All personnel involved in the animal experiments had prior training and extensive experience in the handling and care of laboratory animals, consistent with institutional and national guidelines. Although no additional special training was conducted specifically for this study, all procedures were performed by qualified staff to ensure animal welfare and compliance with ethical standards.** |

***Definition of a humane endpoint**

A humane endpoint is an experimental endpoint at which animals are euthanized when they display early markers associated with death or poor prognosis of quality of life, or specific signs of severe suffering or distress. Humane endpoints are used as an alternative to allowing such conditions to continue or progress to death following the experimental intervention (“death as an endpoint”), or only euthanizing animals at the end of an experiment. Before a study begins, researchers define the practical observations or measurements that will be used during the study to recognize a humane endpoint, based on anticipated clinical, physiological, and behavioral signs. These may include, for instance, body temperature or weight changes, tumor size or appearance, abnormal behaviors, pathological changes, ruffled fur, reduced mobility, body posture, or expression of specific body fluid markers. Please see the NC3Rs guidelines for more information.

**ARRIVE Guidelines**

*PLOS ONE* encourages authors to follow the [Animal Research: Reporting of In Vivo Experiments (ARRIVE) guidelines](http://www.nc3rs.org.uk/arrive-guidelines) for all submissions describing laboratory-based animal research and to upload a completed [ARRIVE Guidelines Checklist](http://www.nc3rs.org.uk/sites/default/files/documents/Guidelines/NC3Rs%20ARRIVE%20Guidelines%20Checklist%20%28fillable%29.pdf) to be published as supporting information.
